# UTE MRI technical developments and applications in osteoporosis: a review

**DOI:** 10.3389/fendo.2025.1510010

**Published:** 2025-02-06

**Authors:** Soo Hyun Shin, Hee Dong Chae, Arya Suprana, Saeed Jerban, Eric Y. Chang, Lingyan Shi, Robert L. Sah, Jeremy H. Pettus, Gina N. Woods, Jiang Du

**Affiliations:** ^1^ Department of Radiology, University of California, San Diego, San Diego, CA, United States; ^2^ Department of Radiology, Seoul National University Hospital, Seoul, Republic of Korea; ^3^ Department of Bioengineering, University of California, San Diego, San Diego, CA, United States; ^4^ Radiology Service, Veterans Affairs San Diego Healthcare System, San Diego, CA, United States; ^5^ Department of Medicine, University of California, San Diego, San Diego, CA, United States

**Keywords:** UTE, MRI, contrast mechanism, quantitation, osteoporosis

## Abstract

Osteoporosis (OP) is a metabolic bone disease that affects more than 10 million people in the USA and leads to over two million fractures every year. The disease results in serious long-term disability and death in a large number of patients. Bone mineral density (BMD) measurement is the current standard in assessing fracture risk; however, the majority of fractures cannot be explained by BMD alone. Bone is a composite material of mineral, organic matrix, and water. While bone mineral provides stiffness and strength, collagen provides ductility and the ability to absorb energy before fracturing, and water provides viscoelasticity and poroelasticity. These bone components are arranged in a complex hierarchical structure. Both material composition and physical structure contribute to the unique strength of bone. The contribution of mineral to bone’s mechanical properties has dominated scientific thinking for decades, partly because collagen and water are inaccessible using X-ray based techniques. Accurate evaluation of bone requires information about its components (mineral, collagen, water) and structure (cortical porosity, trabecular microstructure), which are all important in maintaining the mechanical integrity of bone. Magnetic resonance imaging (MRI) is routinely used to diagnose soft tissue diseases, but bone is “invisible” with clinical MRI due to its short transverse relaxation time. This review article discusses using ultrashort echo time (UTE) sequences to evaluate bone composition and structure. Both morphological and quantitative UTE MRI techniques are introduced. Their applications in osteoporosis are also briefly discussed. These UTE-MRI advancements hold great potential for improving the diagnosis and management of osteoporosis and other metabolic bone diseases by providing a more comprehensive assessment of bone quantity and quality.

## Introduction

Osteoporosis (OP) is a progressive bone disease that is characterized by low bone mass and structural deterioration (1). Fractures are among the most dramatic sequelae. OP affects more than 10 million people in the USA and causes more than two million fractures, with an annual cost estimated at about $19 billion (2). The need for focused preventive strategies has become a major public health priority.

The current standard technique for assessing bone fracture is dual-energy X-ray absorptiometry (DXA), which can only provide information on bone mineral density (BMD) (3). However, the majority of fractures cannot be explained by BMD alone. Bone is a composite material consisting of, by volume, mineral (~43%), organic matrix (~35%), and water (~22%) (4, 5). While bone mineral provides stiffness and strength (6), collagen provides ductility and the ability to absorb energy before fracturing (7), and water contributes to viscoelasticity and poroelasticity (8). These bone components are arranged in a complex hierarchical structure (9). Both material composition and physical structure contribute to the unique strength of bone. The contribution of mineral to bone’s mechanical properties has dominated scientific thinking; however, accurate evaluation of bone requires information about its components (mineral, collagen, water) and structure (cortical porosity, trabecular microstructure), which are all important in maintaining the mechanical integrity of bone (10).

Unfortunately, no single modality can evaluate all bone components and structures. DXA can only measure areal BMD without information about bone collagen, water, and bone microstructure. Computed tomography (CT) can measure volumetric BMD and capture bone structure without information about bone collagen and water (11). Conventional CT has a spatial resolution that is too low to evaluate cortical porosity. High-resolution peripheral quantitative CT (HR-pQCT) can assess bone porosity but cannot resolve smaller pores (e.g., pores with diameters less than 83 µm) (12, 13). Micro CT (µCT) is the reference standard for evaluating cortical porosity but cannot be used for *in vivo* applications (14). Magnetic resonance imaging (MRI) is routinely used to diagnose soft tissue diseases, but bone is “invisible” with clinical MRI due to its short transverse relaxation time (15, 16). This review paper aims to summarize the recent developments in ultrashort echo time (UTE) MRI techniques for direct imaging of bone.

## Materials and methods

This narrative review was conducted to synthesize the most relevant advancements and applications of UTE MRI, particularly focusing on the authors’ contributions and other key studies in the field. The UTE-type sequences include two-dimensional (2D) and 3D UTE (15–28) zero echo time (ZTE) (25–36), pointwise encoding time reduction with radial acquisition (PETRA) (37–39), Cartesian variable TE (vTE) (40), water- and fat-suppressed proton projection MRI (WASPI) (41), sweep imaging with Fourier transformation (SWIFT) (42), hybrid acquisition-weighted stack of spirals (AWSOS) (43), ramped hybrid encoding (RHE) (44), and Looping Star (45). A simple search on Pubmed shows more than 600 papers on direct imaging of bone using the various UTE-type sequences. It is difficult to summarize all the published articles in this review. The selection of articles was primarily guided by the authors’ expertise and their understanding of the pivotal developments in UTE MRI research.

In conventional MRI, bone produces near zero signal, leading most clinicians to rely on plain radiography or CT as the primary modality for bone evaluation. The lack of detectable signals can be mainly attributed to the bone’s short mean apparent transverse relaxation time (T2) or apparent transverse relaxation time (T2*) components. T2 or T2* relaxation time refers to the time constant that describes the rate at which excited protons lose phase coherence due to interactions with surrounding tissues in MRI, with short T2* values indicating a rapid decay of transverse magnetization. Long T2* tissues retain a detectable signal level at the time of the measurement of the MR signal, allowing them to remain visible in conventional pulse sequences. In contrast, short T2* tissues such as bone, tendons, ligaments, and menisci lose most of their signal before spatial encoding, resulting in undetectable signals during signal acquisition, making these tissues appear dark or “invisible” on conventional MRI scans.

For simplicity, T2* values can be categorized into five groups: <0.01 ms (supershort), 0.01–1 ms (ultrashort), 1–10 ms (short), 10–100 ms (intermediate), and 100–4000 ms (long) (16). Echo time (TE) is the interval between the delivery of the RF pulse and the measurement of the MR signal. It determines the time the system waits before measuring the signal. A general rule is that the effective TE should match the T2* of the tissue for optimal detectability. Recent advances in hardware have enabled gradient-recalled echo (GRE) sequences with much reduced TEs to capture signals from short T2 tissues. However, conventional sequences, such as fast spin echo (FSE) and GRE, cannot produce echo times shorter than 1 ms on clinical MRI systems. Therefore, tissues with ultrashort T2 values, such as bone, require specialized techniques for effective signal detection.

Recently, a group of UTE-type sequences, including 2D and 3D UTE, ZTE, PETRA, vTE, WASPI, SWIFT, AWSOS, RHE, and Looping Star sequences, with nominal TEs of 0.1 ms or less have been developed to directly image short-T2 tissues (15–45). While a short TE is essential for imaging bone, it alone is insufficient due to the low proton density in bone (i.e., ~22% water by volume in normal bone). Effective suppression of long-T2 signals is crucial for achieving high-contrast images of bone. Quantitative UTE imaging can provide valuable insights into bone structure and components. In the next section, we will review technical developments in morphological and quantitative UTE imaging of bone. Their applications in osteoporosis will also be briefly discussed.

## Results

### Part I: technical developments in morphological UTE MRI

With the UTE technique, bone signal with an ultrashort transverse relaxation time can be captured. However, UTE MRI is primarily T1-weighted with negative contrast between bone and neighboring musculoskeletal tissues, such as muscle and marrow fat, which have far higher proton densities than that of bone. A key issue for high contrast morphological imaging of bone is the efficient suppression of long T2 signals from surrounding muscle and marrow fat (46). Different contrast mechanisms have been developed for this purpose.

#### UTE with echo subtraction

**Figure 1:**
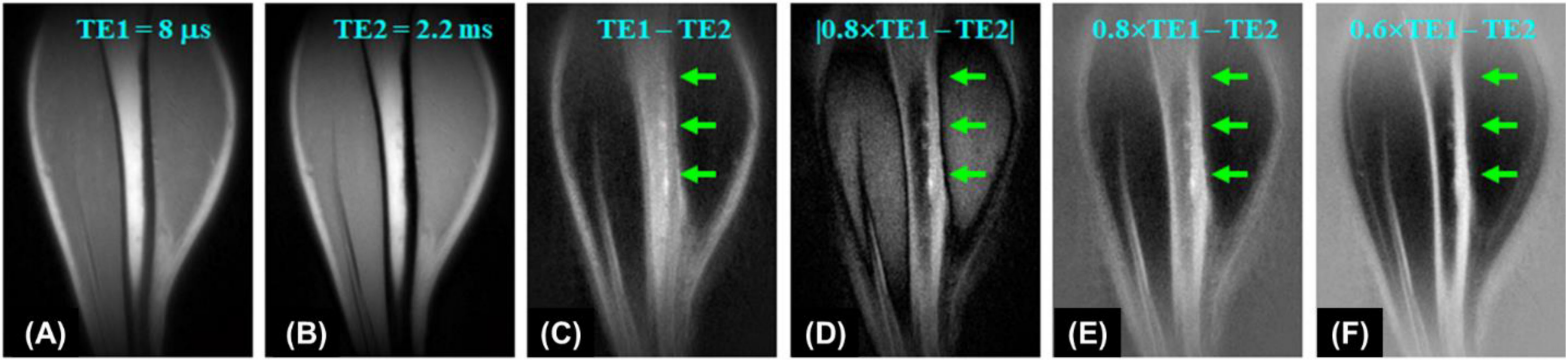
3D UTE imaging of the tibia of a volunteer with dual TEs of 8 µs **(A)** and 2.2 ms **(B)**. Subtraction of the second echo (TE = 2.2 ms) from the first one (TE = 8 µs) shows limited contrast for cortical bone due to a high signal from marrow fat **(C)**. Higher bone contrast is achieved by scaling down the first echo UTE image by a factor of 0.8 and using absolute pixel intensity in the subtraction image **(D)**. Bone contrast can be further enhanced by allowing negative signal intensity in long-T_2_ tissues **(E, F)**. From Ref. (46), with permission.

One basic approach to enhancing contrast in UTE imaging is subtracting two images acquired at distinct echo times (TEs). In the dual-echo UTE imaging technique with echo subtraction, bone contrast is acquired by subtracting a second echo image from a first echo image which is equivalent to T2 bandpass filtering (19). Signals from long T2 tissues experience minimal decay by the time of the second echo, while the signal from bone undergoes significant decay by the time of the second echo. As a result, long T2 tissues show a high signal in the second echo, while bone shows a signal void. Subtraction of the second echo image from the first echo image leads to suppression of long T2 signals, leaving bone signal minimally unaffected, creating high contrast for cortical bone. Rescaled subtraction (46), where the first UTE free induction decay (FID) image is scaled down prior to subtraction to lower signal from long-T2 tissues in the first compared to the second echo, works more efficiently in creating high positive contrast for short-T2 species, especially cortical bone, which has a much lower mobile proton density than surrounding muscle or fat. **Figure 1** shows an example of 3D dual-echo UTE imaging with rescaled subtraction applied to the tibia of a healthy volunteer. Conventional 3D UTE imaging provides a relatively high signal but negative contrast for the tibia (**Figure 1A**). Regular echo subtraction presents a positive contrast between bone and muscle, but a negative contrast between cortical bone and fat, as fat also has a short T2* (**Figure 1C**). The contrast between bone and fat/muscle increases using the rescaled subtraction technique (**Figures 1D–F**). However, subtraction techniques are sensitive to patient motion, which can cause misalignment between the source images and result in artifacts.

#### UTE with long T2 saturation

Preparation pulses can be employed to selectively suppress signals from long T2 components, improving contrast by allowing better visualization of short T2 tissues (47–49). In UTE imaging with long T2 saturation, saturation pulses are used to suppress the signals from long T2 tissues, such as muscle and bone marrow fat, which typically produce higher signals than bone. For example, a 90° pulse with a relatively long duration and a low amplitude can flip the longitudinal magnetization of long T2 tissues into the transverse plane, where a large spoiling gradient can subsequently dephase the transverse magnetization (48). In comparison, bone magnetization is barely excited by this long saturation pulse as the decay rate of bone exceeds the excitation rate. Therefore, a long 90° pulse can be used with a large spoiling gradient to suppress long T2 tissues, leaving bone to be subsequently detected by UTE data acquisition. T2 selective RF excitation (TELEX) can be used to increase bone contrast (47). Dual-band long-T2 suppression pulses further improve the suppression of signals from muscle and fat (49). However, residual signals from muscle and marrow fat due to B1 and B0 inhomogeneities may still compromise bone contrast.

#### UTE with off-resonance saturation

Off-resonance saturation with subtraction can generate contrast for short T2 components by utilizing the broader absorption line shape of short T2 tissues, such as bone, compared to long T2 tissues like muscle or fat, making them more sensitive to off-resonance RF radiation (50). UTE imaging with off-resonance saturation contrast (UTE-OSC) employs a high-power saturation pulse placed a few kHz off the water peak to preferentially saturate signals from bone, leaving long T2 muscle and fat signals largely unaffected (50). Subtraction of UTE images with off-resonance saturation from basic UTE images can effectively suppress signals from muscle and fat, creating high bone contrast.

#### UTE with adiabatic inversion

**Figure 2:**
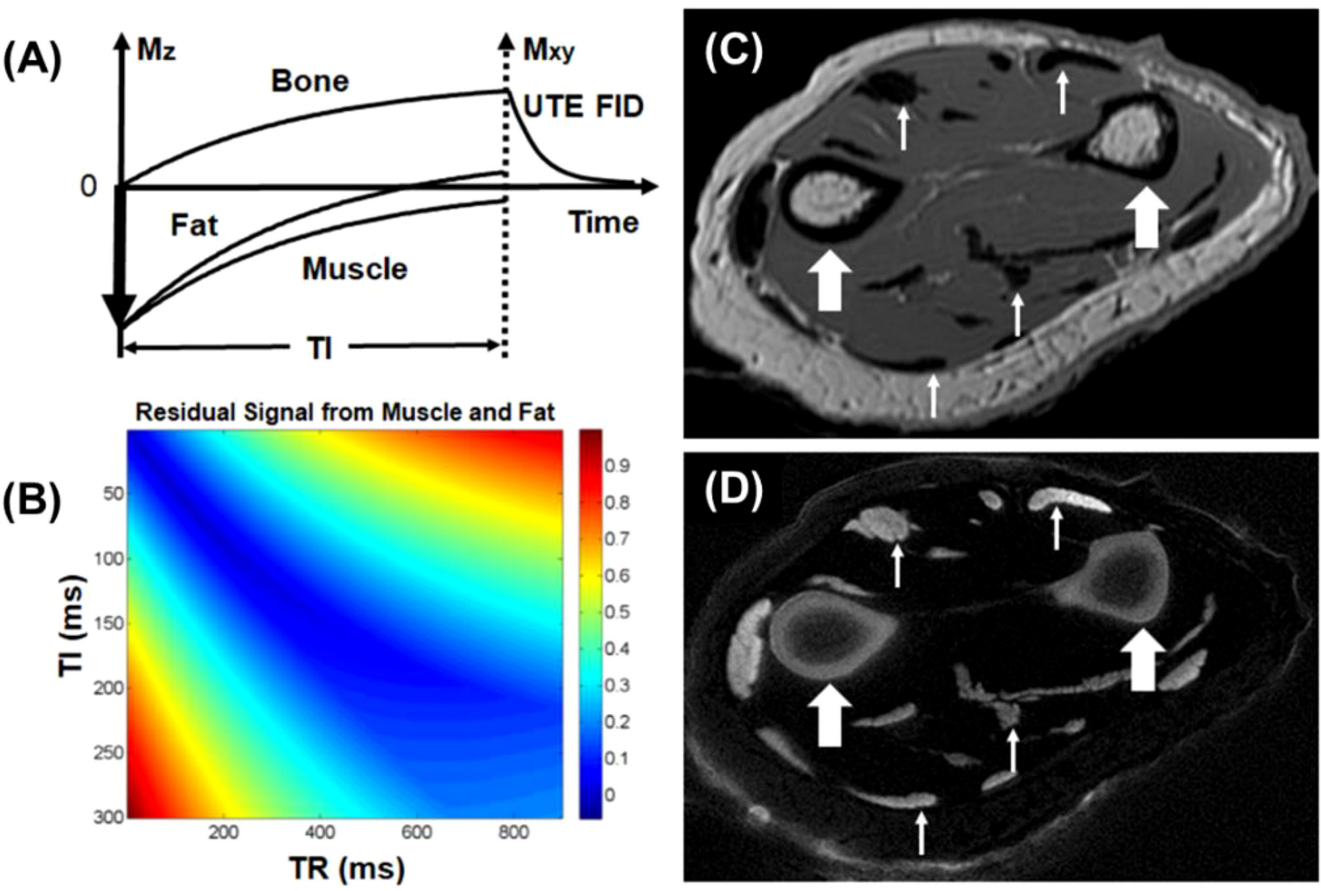
The IR-UTE sequence inverts the longitudinal magnetizations of long T2 muscle and fat with a long adiabatic inversion pulse (duration = 8.64 ms) **(A)**. The longitudinal magnetization of bone is largely saturated, recovers during TI, and is subsequently detected by the UTE data acquisition **(B)**. Clinical FSE imaging of the forearm shows pure signal void for cortical bone (thick arrows), tendons, and aponeuroses (thin arrows) **(C)**. The IR-UTE sequence shows high signal and contrast for cortical bone (thick arrows) and other short T2 tissues (thin arrows) **(D)**. From Ref. (21), with permission.

One limitation of saturation techniques that utilize hard RF pulses is their sensitivity to B0 and B1 inhomogeneities, making them less robust compared to adiabatic inversion (18). The adiabatic inversion recovery UTE (IR-UTE) contrast mechanism employs a long adiabatic inversion pulse to invert the longitudinal magnetizations of long-T2 water (e.g., muscle) and long-T2 fat (18, 21, 24, 51). The duration of the adiabatic inversion pulse is much longer than bone T2* (18). As a result, the longitudinal magnetizations of muscle and marrow fat are fully inverted, while the bone magnetization is not inverted but largely saturated by the long adiabatic inversion pulse (51). The UTE data acquisition starts at an inversion time (TI) adjusted so that the inverted long T2 magnetizations approach the null points, leaving the uninverted bone magnetization being selectively detected by UTE data acquisition. The adiabatic inversion pulse has a relatively broad spectral bandwidth, thereby insensitive to B1 and B0 inhomogeneities. The IR-UTE technique allows uniform inversion of long T2 magnetizations, providing robust high contrast imaging of bone (18, 21). **Figure 2** shows representative IR-UTE images of cortical bone in the forearm, which is depicted with excellent image contrast but invisible with conventional clinical FSE sequences.

#### UTE with double adiabatic inversion

**Figure 3:**
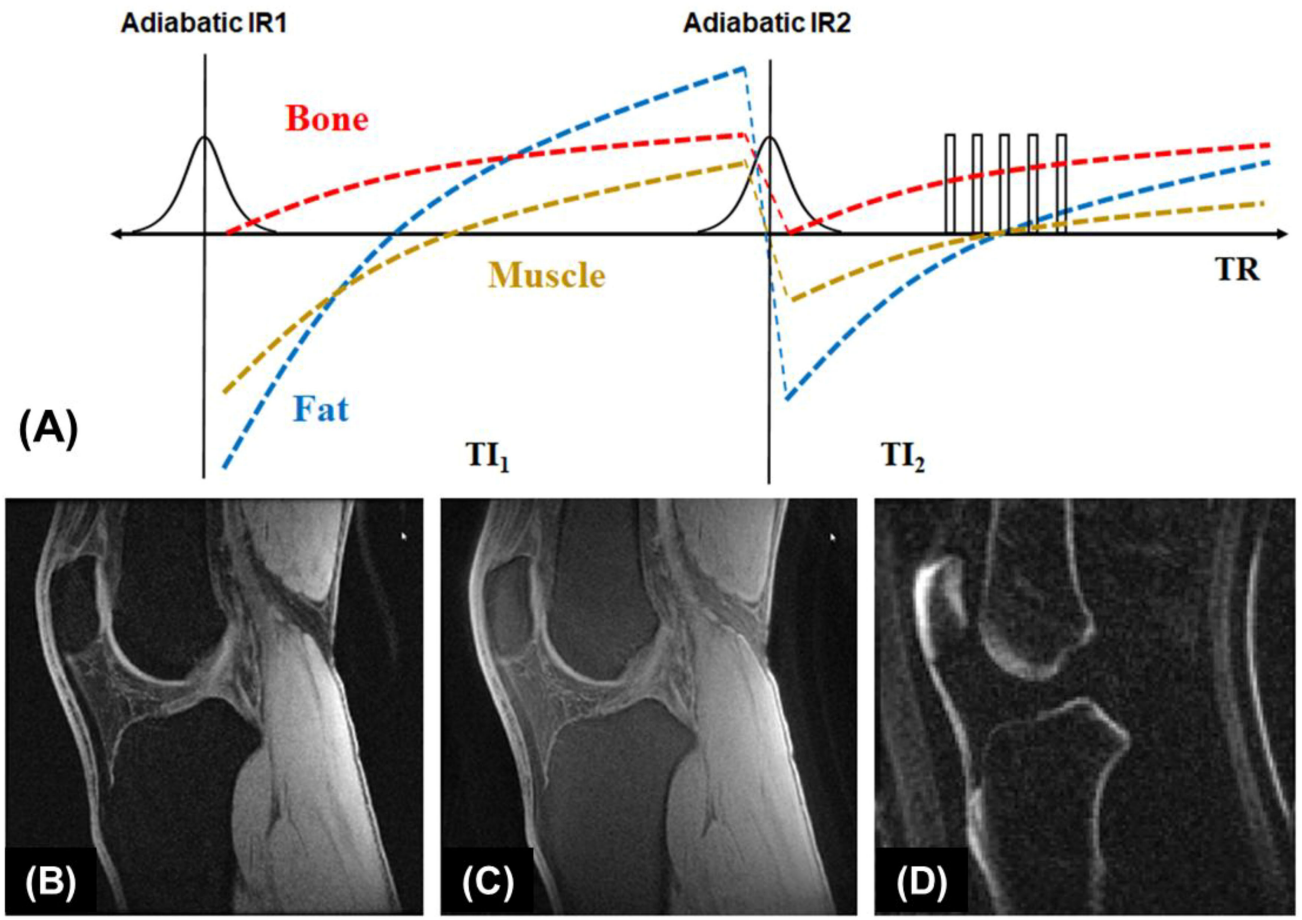
The double-IR-UTE sequence employs two identical adiabatic inversion pulses for simultaneous suppression of long T2 muscle and fat with different T1s, followed by 3D UTE data acquisition to produce high contrast imaging of bone **(A)**. The knee joint of a 31-year-old volunteer was subject to clinical GRE **(B)**, fat-saturated UTE **(C)**, and double-IR-UTE **(D)** imaging. The double-IR-UTE sequence shows excellent suppression of muscle and fat, providing high contrast for the patellar tendon and cortical bone **(D)**. From Ref. (55), with permission.

A single inversion pulse can reduce the signal from fat and long T2* components (such as muscle) by up to 80% (21). However, using dual inversion pulses allows for the complete nulling of both, providing more effective signal suppression (52–54). The double adiabatic inversion recovery UTE sequence (double-IR-UTE) employs two identical adiabatic inversion pulses (duration of ~6 ms) with the same center frequency to sequentially invert the longitudinal magnetizations of long T2 species, followed by multispoke UTE data acquisition (55). The two adiabatic inversion pulses are applied with pre-defined inversion times TI1, which is the time between the centers of the two adiabatic inversion pulses, and TI2, which is the time from the center of the second adiabatic inversion pulse to the center spoke of the multispoke acquisition. Robust long T2 suppression can be achieved by timing the center spoke at the null point. Long T2 transverse magnetizations acquired before the null point are of opposite polarity to those acquired after the nulling point, leading to cancellation in the regridding process during image reconstruction and, therefore, efficient suppression of long T2 signals from muscle and marrow fat. Bone magnetization is not inverted but saturated by the two long adiabatic inversion pulses, recovers after the second TI2, and is subsequently detected by UTE data acquisition. The advantage of double-IR-UTE is the robust suppression of long T2 tissues with a broad range of T1s, such as fat and muscle, which can be nulled simultaneously using specific combinations of TI1 and TI2. The double-IR-UTE sequence is insensitive to inhomogeneities in the B1 and B0 fields due to the use of adiabatic inversion pulses with relatively broad spectral bandwidths. **Figure 3** shows double-IR-UTE imaging of the knee joint in a healthy volunteer, which shows high signal from short- and ultrashort-T2 species, such as the patellar tendon and cortical bone.

#### UTE with relaxation-parameter contrast

**Figure 4:**
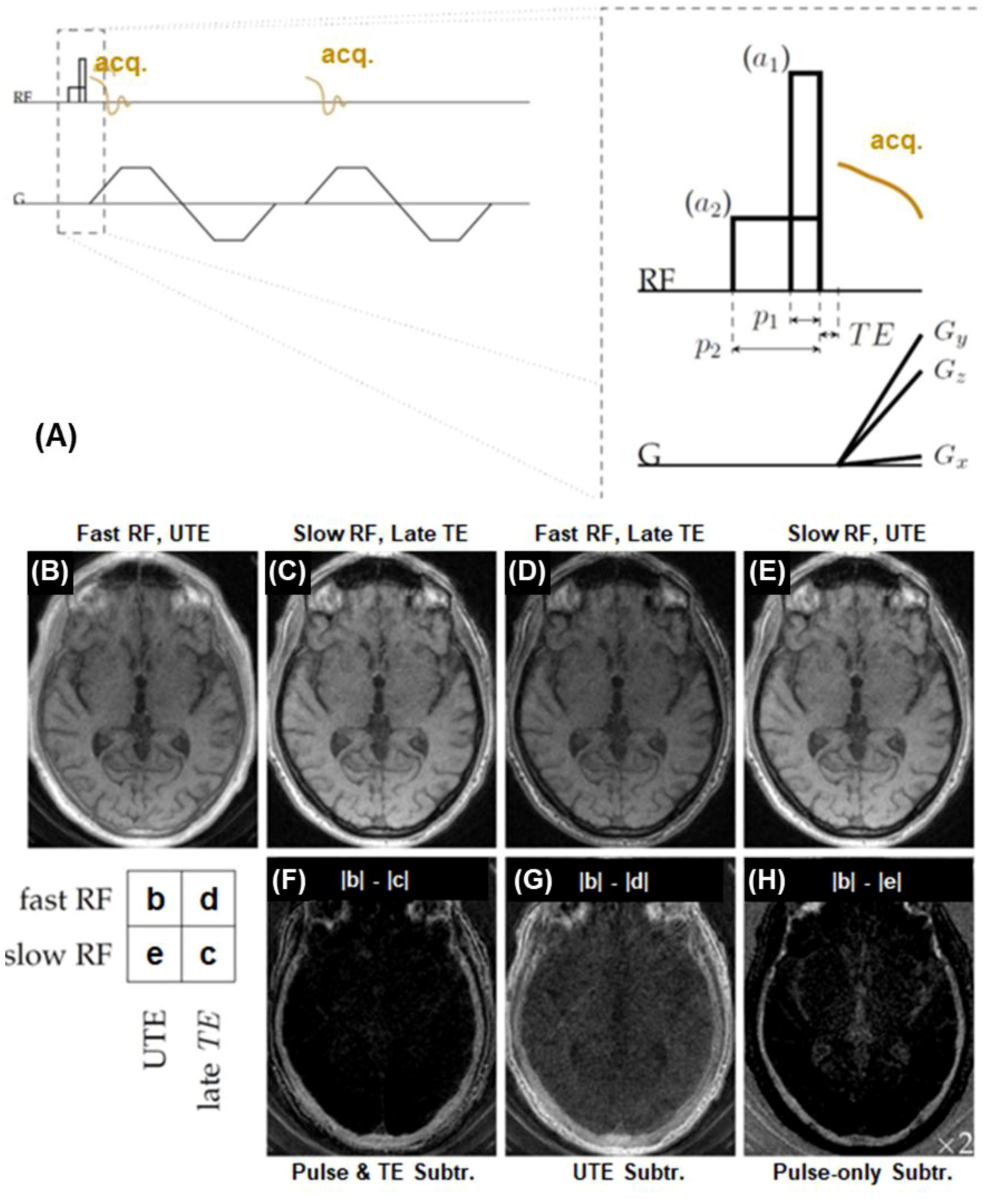
Bone imaging with the relaxation-parameter contrast mechanism, which is based on two hard RF pulses with different durations but equal pulse areas to generate T_2_-selective excitation **(A)**. The contrast mechanism can be combined with single or dual-echo UTE data acquisition using two RF amplitudes (a_1_ and a_2_) and pulse durations (p_1_ and p_2_) with equal pulse areas. An example is shown on a volunteer’s skull, including UTE with a short RF pulse of 24.47 μT and a TE of 34 µs **(B)**, UTE with a long RF pulse of 1.53 μT and a TE of 2.0 ms **(C)**, UTE with a short RF pulse and a longer TE of 2.0 ms **(D)**, and UTE with a long RF pulse and a TE of 34 µs **(E)**. The difference image (|b|-|c|) **(F)** depicts cortical bone more specifically than the conventional UTE subtraction difference image (|b|-|d|) **(G)**, and captures more bone signal than the pulse-only difference image (|b|–|e|) **(H)**. From Ref. (56), with permission.

UTE data acquisition can be combined with relaxation-parameter contrast (56). UTE with relaxation parameter contrast and subtraction exploits the sensitivity of bone proton magnetization to both T2 and RF pulse duration. Excitation pulse parameters are selected to determine the extent of concurrent relaxation and excitation. The RF pulse duration and amplitude can be changed to adjust the relaxation dependence of bone contrast. To selectively detect signals from magnetization within a specific range of T2 values, two RF pulse durations are chosen so that the sensitivity transition between them brackets the range of interest. Two UTE datasets with similar imaging parameters but different RF excitation pulses are acquired. Bone contrast is created by subtraction of the two UTE images, as shown in **Figure 4**.

#### UTE with dual-RF and dual-echo (DURANDE)

The 3D DURANDE UTE sequence and bone-selective image reconstruction have been proposed for rapid bone imaging (57). This technique acquires two dual-echo UTE datasets following short and long RF pulses, with encoding gradients varying continuously along the entire pulse train to halve the total imaging time. The DURANDE UTE sequence employs two rectangular RF pulses (RF1 and RF2), differing in duration and amplitude but having the same pulse area applied alternately in successive TR periods along the entire pulse train. Two echoes at a short TE and a long TE are collected from the beginning of the gradient ramp-up within each TR. As a result, four echoes are produced and combined via a view-sharing approach to generate two independent k-space datasets during image reconstruction. Accelerated UTE bone imaging can be achieved by using the sparsity of bone voxels in the corresponding subtraction images.

#### Short TR adiabatic inversion recovery UTE MRI of trabecular bone

**Figure 5:**
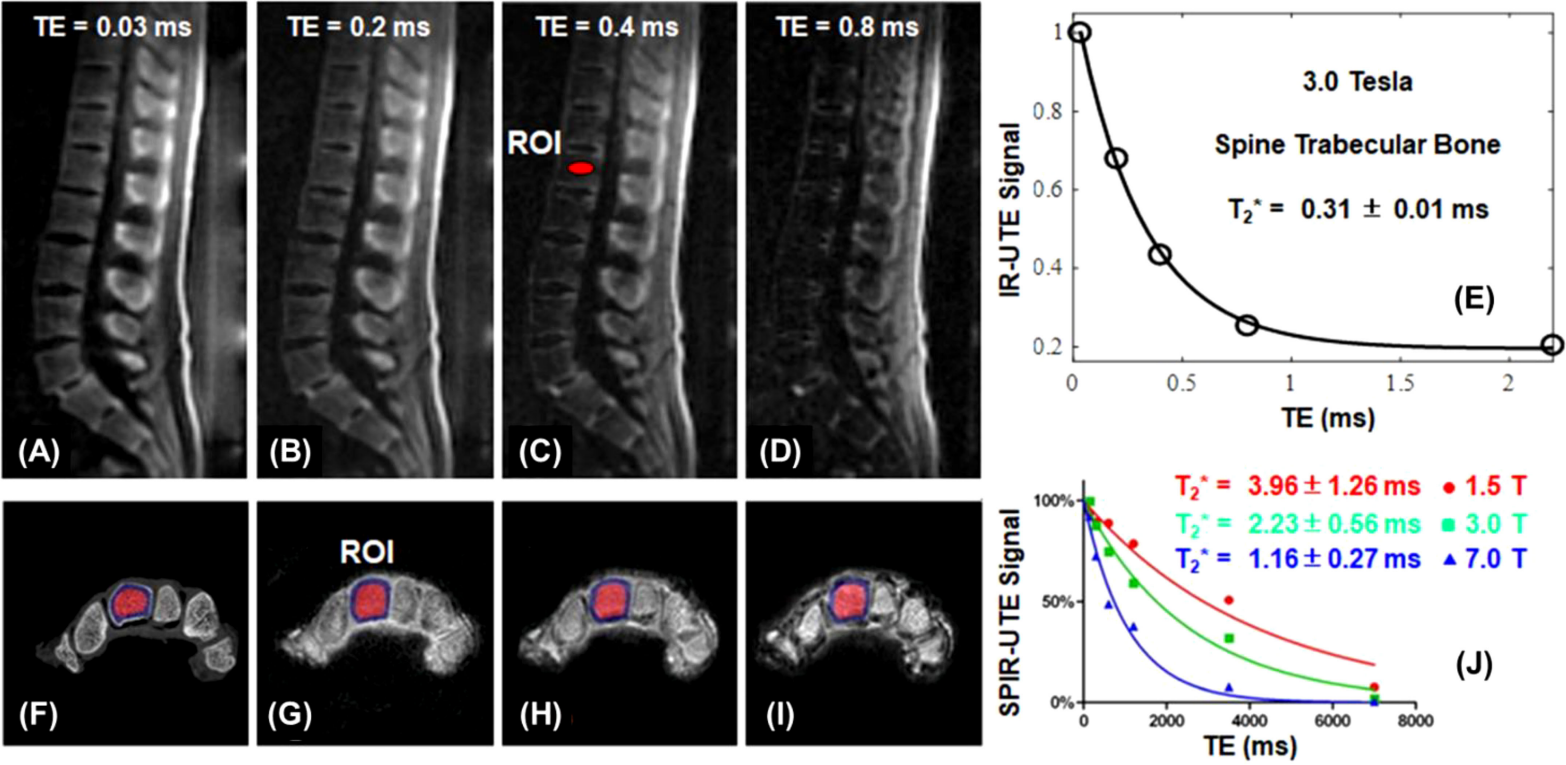
STAIR-UTE imaging of trabecular bone in the spine of a 36-year-old male volunteer with TEs of 0.032 ms **(A)**, 0.2 ms **(B)**, 0.4 ms **(C)**, and 0.8 ms **(D)** at 3T, and the single-component T_2_* fitting **(E)**. µCT **(F)** and SPIR-UTE imaging of trabecular bone in the fingers at 1.5 T **(G)**, 3.0 T **(H)**, 7.0 T **(I)**, and the corresponding single component T_2_* fitting **(J)**. STAIR-UTE imaging of trabecular bone in the spine shows a short-T_2_* of 0.31 ± 0.01 ms at 3.0 T, while SPIR-UTE imaging of trabecular bone in the fingers shows short-T_2_* values of 1.16 ± 0.27 ms at 7.0 T, 2.23 ± 0.56 ms at 3.0 T, and 3.96 ± 1.26 ms at 1.5 T, respectively. From Refs. (60, 61), with permission.

In STAIR-UTE, 3D IR-UTE data are acquired with a short TR and a high flip angle within specific absorption rate (SAR) limits for clinical imaging (58–60). The short TR and TI combination is selected to achieve robust suppression of long-T2 muscle and marrow fat regardless of their different T1 values. Multiple spokes are acquired for efficient volumetric imaging of cortical and trabecular bone (60). The STAIR-UTE sequence is more efficient than other UTE or ZTE techniques, such as the spectral presaturation with IR UTE (SPIR-UTE), in selective imaging of trabecular bone (61). **Figure 5** shows STAIR-UTE images of the spine and SPIR-UTE images of the fingers. The SPIR-UTE images showed T_2_* values of 2.42 ± 0.56 for the capitate, which is much longer than the T_2_* of 0.31 ± 0.01 ms for the trabecular bone of the spine measured on STAIR-UTE images (60, 61), or the T_2_* value of ~0.3 ms for the cortical bone (62). The much longer T_2_* values suggest that SPIR-UTE imaging of the trabecular bone is subject to significant long-T2 signal contamination. In comparison, STAIR-UTE-measured T_2_* values for the trabecular bone are close to those measured for cortical bone, suggesting that bone marrow fat is completely suppressed and only signal from trabeculae is selectively detected in STAIR-UTE imaging (60).

#### UTE on the fat peak for trabecular bone imaging

**Figure 6:**
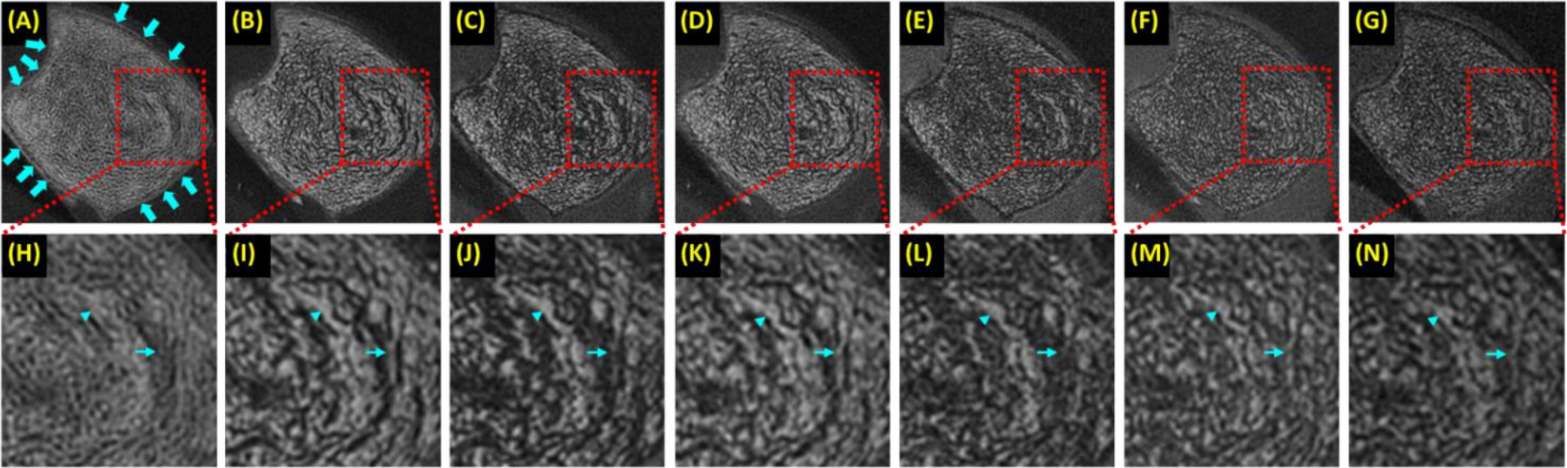
A trabecular bone specimen imaged with 3D UTE on the water peak at TE of 0.03 ms **(A)** and on the fat peak at TEs of 0.03 ms **(B)**, 1.1 ms **(C)**, 2.2 ms **(D)**, 3.3 ms **(E)**, and 4.4 ms **(F)**, and clinical 3D GRE at TE of 4.4 ms **(G)**, with the zoomed regions indicated with the red dashed-line boxes shown in the second row **(H-N)**. UTE images on the water peak show significant chemical shift artifacts, manifesting as blurred trabecular bone structure and ringing artifacts [arrows in **(A)**]. The more significant fat signal loss was observed at longer TEs **(C-F, J-M)** due to the strong susceptibility between bone/marrow interface and at TEs of 1.1 ms **(C, J)** and 3.3 ms **(E, L)** due to fat/water signal cancellation, with both leading to overestimation of trabecular thickness. From Ref. (66) with permission.

Past research has focused on high resolution imaging of marrow to indirectly detect trabecular microstructure (5, 63, 64). Two major challenges exist: the high susceptibility at the marrow/bone interface and the multiple fat peaks, both of which significantly reduce T_2_*, leading to low marrow signal (misclassified as bone) and overestimation of trabecular volume. UTE is insensitive to T_2_* shortening. However, UTE employs non-Cartesian radial sampling, which is sensitive to chemical shift artifacts (65). UTE imaging on the fat peak resolves this issue (66). Bone is off-resonance in fat-centered imaging, but it has a much lower signal than marrow, and the off-resonance artifact is negligible. **Figure 6** shows UTE and clinical GRE imaging of a trabecular bone sample from a 65-year-old male donor. UTE on the water peak shows strong chemical shift artifacts, which are significantly reduced in UTE imaging on the fat peak. Trabecular bone thickness is overestimated at longer TEs (e.g., TE = 1.1, 2.2, 3.3, or 4.4 ms) or with the clinical GRE sequence. UTE imaging on the fat peak is expected to perform even better in older osteoporotic or diabetic patients who typically have a higher fat fraction in the marrow.

#### ZTE MRI of cortical bone

ZTE employs a short rectangular pulse excitation followed by readout gradient flat-top sampling to minimize the effective TE (29). A small flip angle (1-2°) is typically used to minimize the dead-time gap, which causes a spherical void in the center of k-space. A variety of approaches have been developed to address this k-space gap and associated low frequency artifacts in the reconstructed images (33). The repetition time (TR) is minimized to speed up data acquisition. Higher receiver bandwidths (62.5-83.3 kHz) are recommended to mitigate chemical shift artifacts. Bias field correction, contrast inversion, and background segmentation are employed for CT-like bone contrast (34–36). The principal difference between ZTE and UTE sequences is the temporal order of setting the spatial encoding gradient and RF excitation (33). UTE offers the freedom to adjust TE, a feature not possible in ZTE imaging. UTE also allows high flip angles, a significant advantage in direct bone imaging using the STAIR contrast mechanism (60). On the other hand, the ZTE sequence acquires k-space data after the readout gradients are fully ramped up, avoiding fidelity issues introduced by gradient ramping (33). ZTE has a shorter effective TE and can detect signal from shorter T2 species. ZTE can be applied in many of the same applications and with many of the same magnetization preparation methods as UTE.

#### Other UTE-type sequences for bone imaging

Many other UTE-type sequences have been developed for bone imaging. These sequences can be combined with each of the above contrast mechanisms for high-contrast imaging of bone. For example, adiabatic inversion recovery-based preparations can be combined with ZTE (29–36), vTE (40), AWSOS (43), RHE (44), and PETRA (37–39) sequences for high contrast imaging of cortical bone and other short-T_2_ tissues, respectively. On-resonance long-T_2_ suppression or off-resonance short-T_2_ saturation can be applied to SWIFT, PETRA, WASPI, RHE, and ZTE sequences to create short-T_2_ contrast. For example, SWIFT with off-resonance saturation has been used to image the interface between cartilage and subchondral bone (67). A systematic study of the above contrast mechanisms combined with ZTE, vTE, WASPI, SWIFT, AWSOS, PETRA, RHE, and Looping Star sequences remains to be investigated, and their SNR and CNR efficiency remains to be compared.

### Part II: technical development in quantitative UTE imaging

Quantitative UTE MRI techniques have been developed to evaluate bone MR relaxation properties such as T1 and T2* relaxation times, and tissue properties such as total water proton density (TWPD), bound water proton density (BWPD), pore water proton density (PWPD), macromolecular proton density (MMPD), magnetization transfer ratio (MTR), susceptibility, and perfusion (24–26).

#### Bone T1 relaxation time

T1 relaxation is a fundamental MR tissue property and describes how fast the longitudinal magnetization recovers to the steady state. Many T1 measurement techniques have been combined with UTE acquisitions to provide accurate T1 measurements of bone, such as saturation recovery UTE (18), inversion recovery UTE (68), UTE with variable repetition time (UTE-VTR) (69), and UTE with variable flip angle (UTE-VFA) methods (70). The UTE-VTR method is sensitive to B1 inhomogeneity. The actual flip angle imaging (AFI) method has been widely used for 3D B1 mapping (71). By combining UTE and AFI techniques, it is possible to use a pair of interleaved UTE acquisitions with a short TR (e.g., 20 ms) and a longer TR (e.g., 100 ms) to produce accurate B1 mapping for bone (69). Furthermore, combining UTE-VTR and UTE-AFI (UTE-AFI-VTR) provides accurate T1 mapping for bone with B1 correction. A short T1 of ~250 ms was reported for cortical bone (69).

#### Bone T2* relaxation time

**Figure 7:**
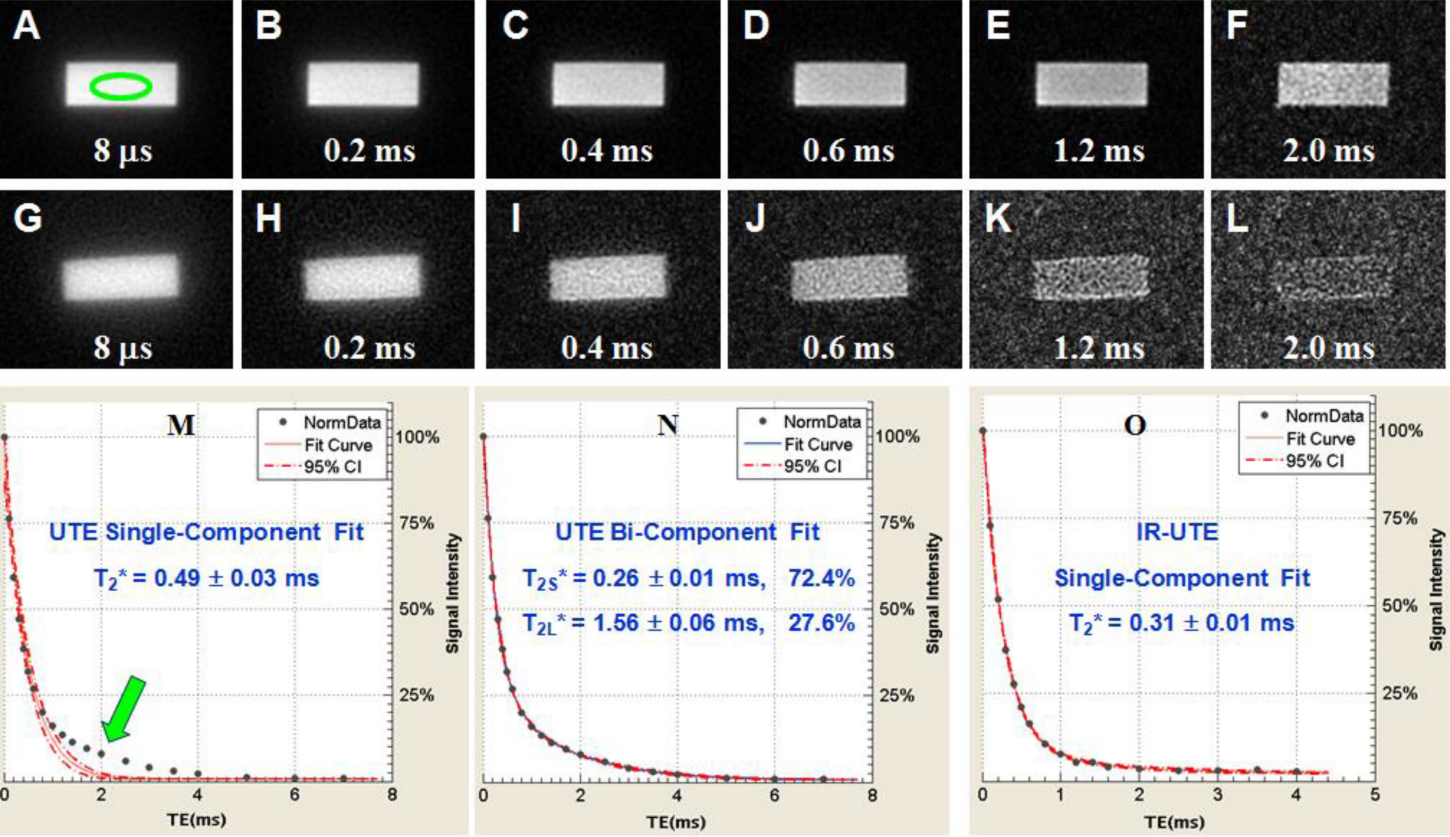
UTE imaging of a sectioned bovine cortical bone with TEs of 8 μs to 2 ms **(A-F)**, IR-UTE with TEs of 8 μs to 2 ms **(G-L)**. Single- **(M)** and bi-component **(N)** fitting suggest two components: bound water with a short T_2_*~0.26 ms and pore water with a T_2_* ~1.56 ms. IR-UTE images show one component with a T_2_* of ~0.31 ms **(O)**, consistent with bound water imaging. From Ref. (77) with permission.

Bone water exists as pore water residing in the macroscopic pores and as loosely bound water attached to the organic matrix (72). UTE sequences can detect pore water with a longer T_2_* of ~3 ms and loosely bound water with an ultrashort T_2_* of ~0.3 ms (62, 73–76). IR-UTE or STAIR-UTE allows partial inversion and nulling of pore water with longer T2*, leaving bound water with ultrashort T2* to be selectively imaged (21, 60, 77). **Figure 7** shows single- and bi-component fitting of UTE and IR-UTE images of a bovine cortical bone sample (77). Excellent bi-component fitting was achieved to show the existence of two distinct water components: bound water with a short T_2_* of 0.26 ms (72.4% by volume) and pore water with a longer T_2_* of 1.56 ms (27.6%). The IR-UTE images show a single component with T_2_* ~0.31 ms, suggesting that pore water is efficiently suppressed and bound water selectively imaged (77).

#### UTE-MT modeling of MMF and exchange rates

**Figure 8:**
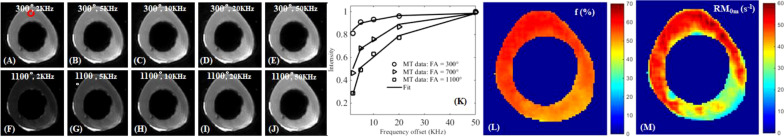
UTE-MT imaging of cortical bone with an MT power of 300° and frequency offsets of 2 kHz **(A)**, 5 kHz **(B)**, 10 kHz **(C)**, 20 kHz **(D)**, 50 kHz **(E)**, and 1100° and 2 kHz **(F)**, 5 kHz **(G)**, 10 kHz **(H)**, 20 kHz **(I)**, 50 kHz **(J)**, and two-pool fitting **(K)** with maps of macromolecular fraction [MMF or f; **(L)**] and exchange rate [RM_0m_; **(M)**]. From Ref. (79) with permission.

There is another group of protons, collagen backbone protons, which have extremely short T2* relaxation times and are invisible with UTE sequences. UTE magnetization transfer (UTE-MT) modeling can measure collagen backbone proton fraction and exchange rates between water and collagen protons (73–75, 78–83). **Figure 8** shows UTE-MT imaging of a bovine bone sample. Excellent two-pool MT modeling and MT parameters mapping were achieved using a Gaussian lineshape (79). The lower half of this bone sample shows increased variations in UTE image signal intensity and MT parameters, suggesting an abnormality that needs further investigation.

#### UTE mapping of water and collagen protons

**Figure 9:**
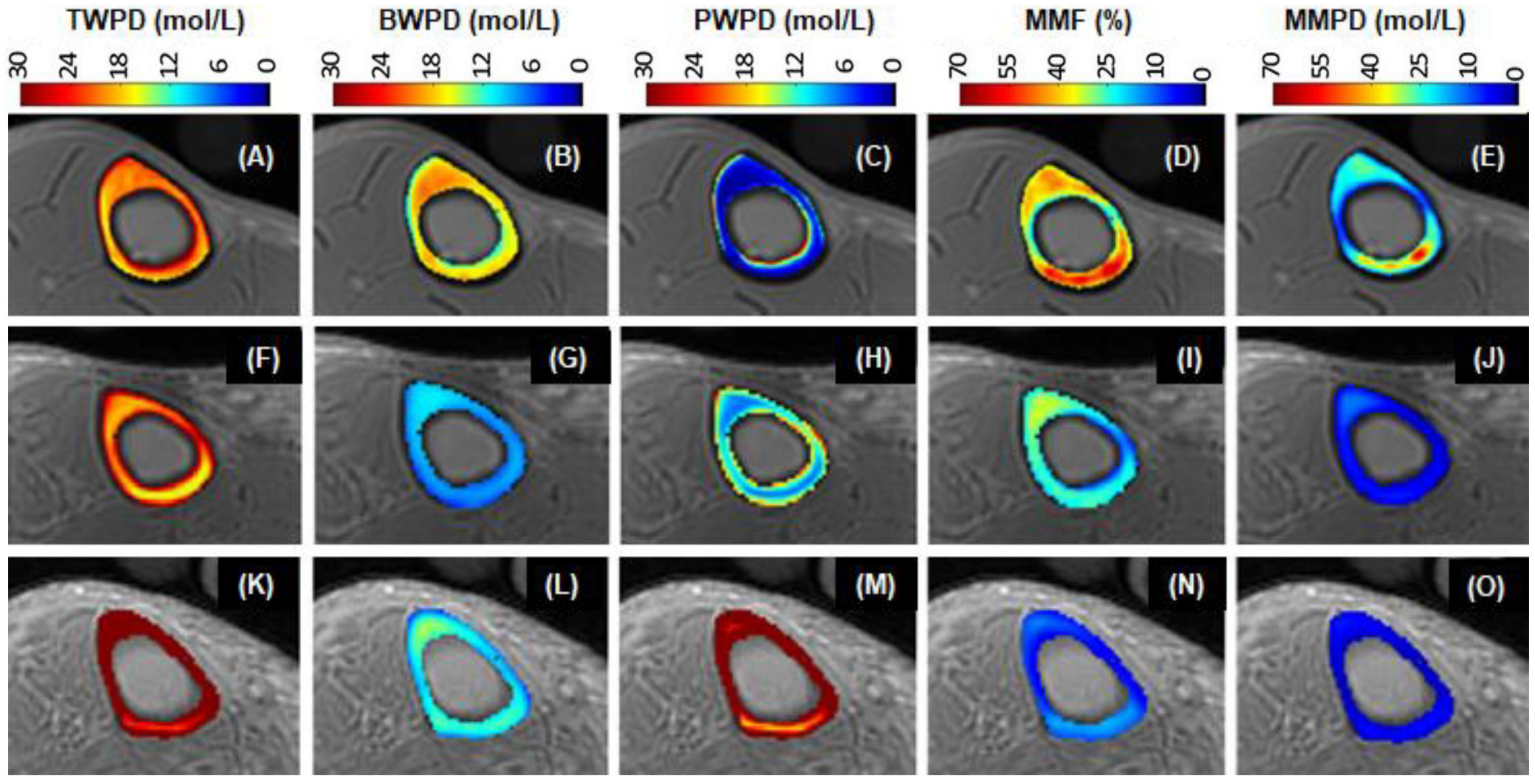
UTE maps of TWPD **(A, F, K)**, BWPD **(B, G, L)**, PWPD **(C, H, M)**, MMF **(D, I, N)**, and MMPD **(E, J, O)** of a 35-year-old healthy (1^st^ row), a 76-year-old osteopenia (2^nd^ row), and a 57-year-old OP (3^rd^ row) females. The OP patient has the highest PWPD but the lowest MMF and MMPD. From Ref. (91) with permission.

UTE sequences can be used to map TWPD, BWPD, PWPD, and MMPD (20–23, 25, 76, 84–91). TWPD can be estimated by comparing the UTE MRI signal of bone with an external reference with known proton density (20, 21, 76, 84–88). BWPD can be measured with IR-UTE or STAIR-UTE, which efficiently suppresses pore water (21, 23, 60). PWPD can be quantified by subtracting bound water from total water. MMPD can be quantified by combining total water proton density with macromolecular fraction (MMF) (91). **Figure 9** shows 3D mapping of TWPD, BWPD, PWPD, and MMPD for tibial midshaft of a 35-year-old healthy female, a 76-year-old female with osteopenia, and a 57-year-old female with OP, respectively (91). The OP patient has higher PWPD but lower MMF and MMPD, consistent with increased porosity and loss of mineral/collagen.

#### UTE quantitative susceptibility mapping

**Figure 10:**
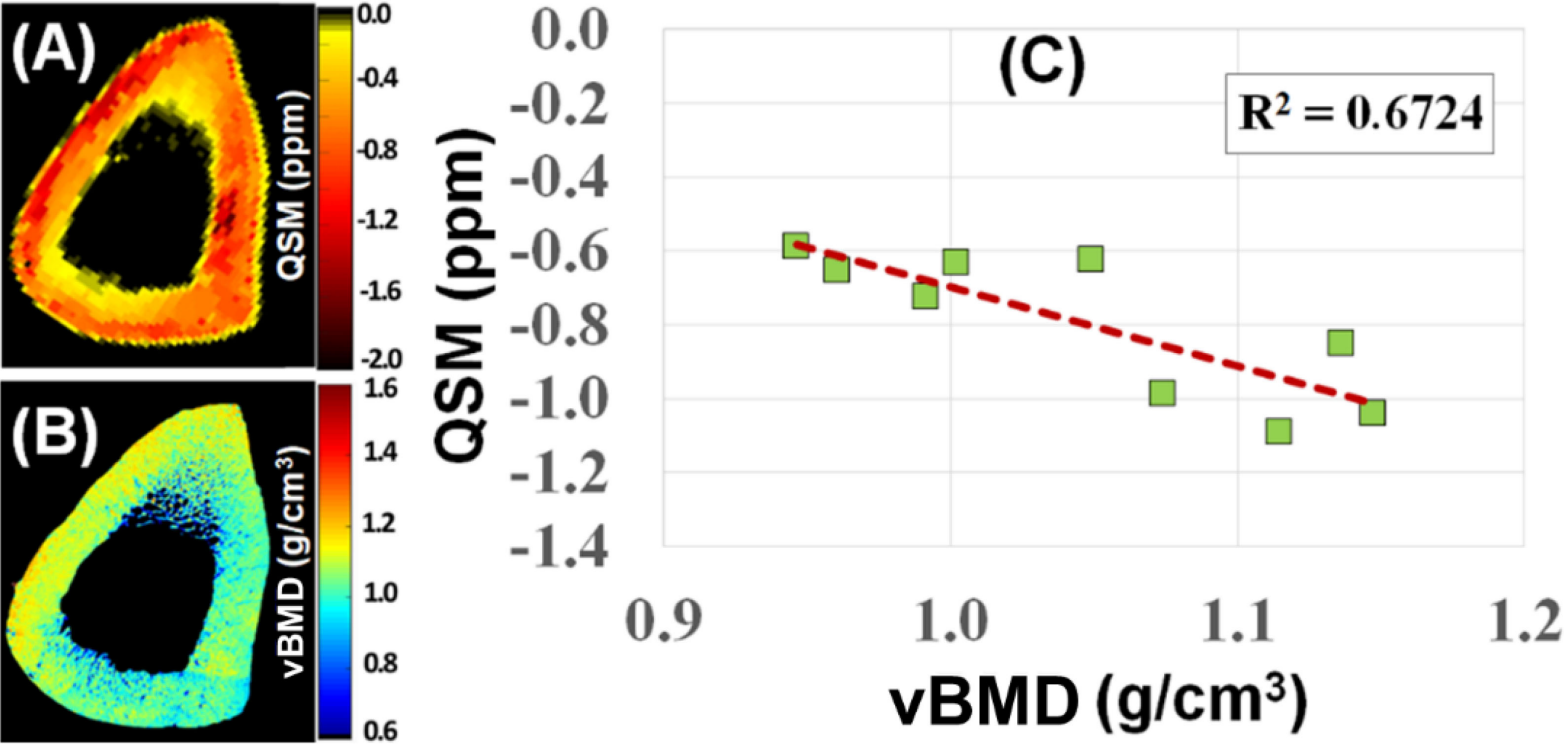
UTE-QSM **(A)** and µCT volumetric BMD (vBMD) **(B)** maps of a human cortical bone sample. A negative correlation (R^2^ = 0.6724) was observed between QSM and vBMD (n=9) **(C)**. From Ref. (98) with permission.

Susceptibility is an important material property. QSM techniques can estimate calcium and iron accumulation in the brain (92). Bone susceptibility is more challenging to measure due to the lack of signal. UTE can detect phase evolution in cortical and trabecular bone. The phase changes with increasing TEs can be used to evaluate bone susceptibility using various algorithms such as Morphology Enabled Dipole Inversion (MEDI) (93). UTE with QSM (UTE-QSM) provides information about bone susceptibility, which is indirectly related to bone mineral (93–98). **Figure 10** shows UTE-QSM and µCT-measured volumetric BMD (vBMD) of a human bone sample, with an excellent linear correlation between QSM and vBMD (n=9). UTE-QSM can reliably evaluate vBMD in cortical bone. Similar results are also observed for trabecular bone.

#### UTE perfusion

There is a close association between bone perfusion and bone remodeling and fracture repair (99–102). Increased cortical bone turnover and inflammation are also associated with increased blood flow (99). There is a strong correlation between bone perfusion and BMD (101, 102). However, the nature of bone makes it difficult to investigate perfusion. The techniques applicable to many soft tissues are difficult or impossible to apply to bone. For example, dynamic contrast-enhanced MRI (DCE-MRI) can be used to study perfusion in various tissues and organs. The technique employs fast T1-weighted images to capture signal changes induced by exogenous intravascular nondiffusible gadolinium-based contrast agents as a function of time. Conventional DCE-MRI can study perfusion in the marrow of trabecular bone (103), but cannot study perfusion in cortical bone due to the lack of detectable signal. Dynamic UTE imaging has been developed to evaluate perfusion in cortical bone (104, 105). A recent study reported dynamic 2D UTE imaging of the tibial midshaft of a 38-year-old healthy volunteer and found ~20% signal enhancement after intravenous gadolinium contrast injection (105). Kinetic analysis demonstrated a K^tran^ of 0.23 ± 0.09 min^-1^ and K_ep_ of 0.58 ± 0.11 min^-1^ for the tibial midshaft of this volunteer. DCE-UTE can potentially be used to evaluate bone remodeling and fracture recovery.

#### Other UTE-type sequences for bone quantification

Bone components (water, collagen, mineral) and microstructure (cortical porosity, trabecular structure) can be qualified by many other UTE-type sequences such as ZTE (25–36), PETRA (37–39), vTE (40), WASPI (41), and SWIFT (42). For example, WASPI has been used to image bone water and the solid matrix of bone (106). SWIFT has been shown to be able to identify the presence and extent of dental caries and fine structures of the teeth, including cracks and accessory canals (107). Furthermore, solid-state 31P MRI can be achieved with UTE-type sequences by focusing on the 31P peak (108). 31P UTE MRI can map phosphorus content, assess bone mineral density, and differentiate between mature and newly remodeled bone (108, 109).

### Part III: applications in OP

#### UTE-measured pore water to assess cortical porosity

**Figure 11:**
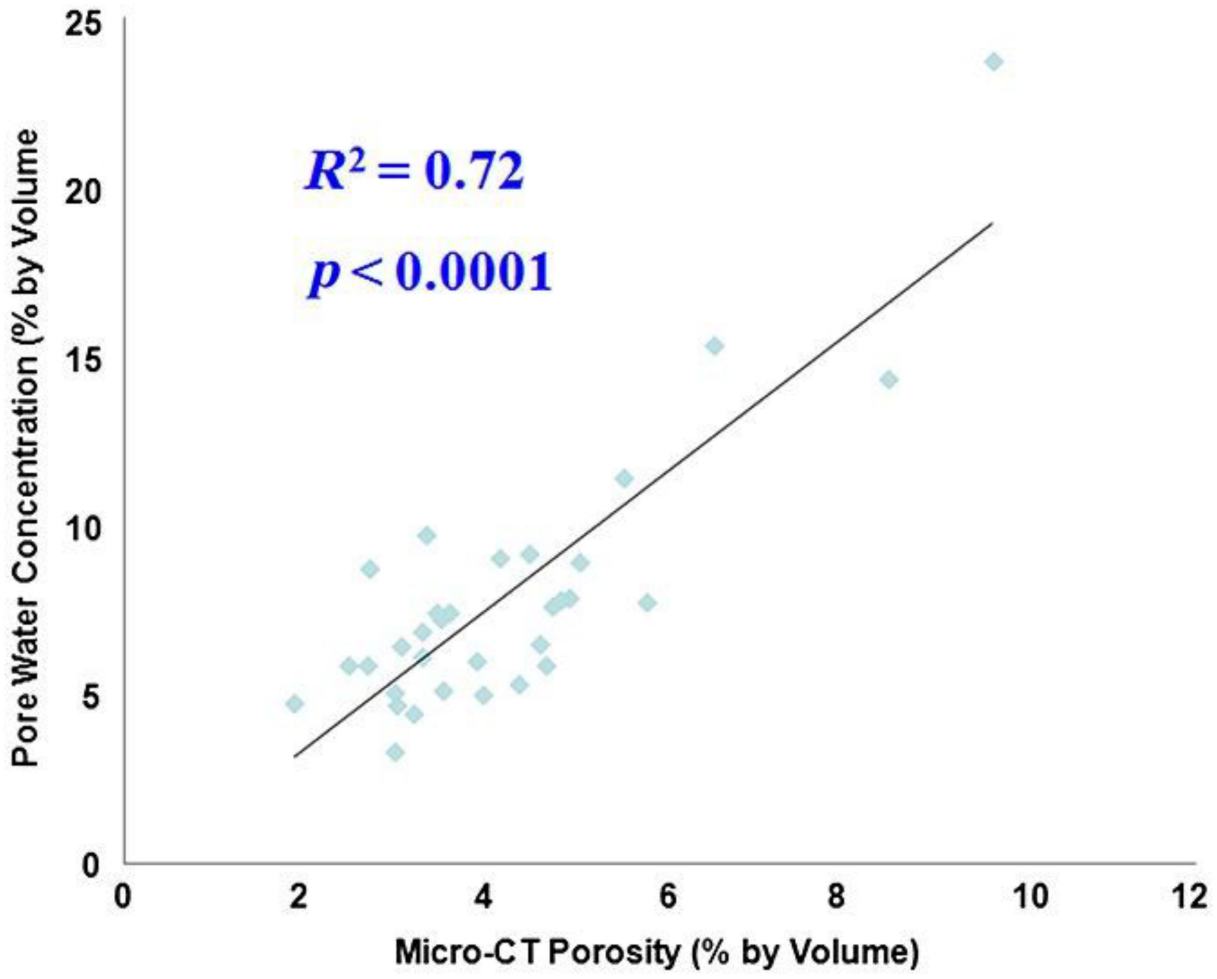
Correlation between UTE-measured pore water concentration and μCT-measured porosity in cadaveric human cortical bone samples (n = 32). A high correlation (R^2^ = 0.72; P < 0.0001) was observed between UTE pore water concentration and μCT porosity, suggesting that UTE sequences can reliably access pore water in cortical bone using a clinical MR scanner. From Ref. (77) with permission.

UTE MRI can be used to measure pore water concentration in cortical bone (23, 76, 84–88). A recent study showed a high correlation (R^2^ = 0.72; P < 0.0001) between μCT porosity and pore water concentration in 32 cadaveric human cortical bone samples (**Figure 11**) (77). Water residing in the microscopic pores of cortical bone is expected to behave more like “free” water with much longer T2* relaxation time than water bound to the organic matrix. Therefore, separating pore water from bound water is easy, allowing accurate pore water mapping without requiring ultrahigh spatial resolution to resolve the small pores. This is confirmed by the high correlation with an R^2^ of 0.72 between μCT porosity and pore water concentration in cortical bone. μCT porosity is consistently lower than pore water content assessed by UTE MRI. Pore water content in cortical bone is also significantly correlated with its mechanical properties (110–112). In another study, UTE MRI, μCT, and histomorphometry were performed on tibial samples from 11 donors. UTE-measured pore water content showed significant correlations (R^2^>0.25) with histomorphometry-based lacunae and small Haversian canals, which are below the detectable range of μCT at 9 μm. The μCT-based porosity showed strong correlations with histomorphometric porosity and pore size when considering all pores or only large pores (R>0.70, P<0.01). Correlations were poor when considering only small pores in histomorphometric analyses (R<0.3) (88). Therefore, pore water in smaller pores can be detected by UTE MRI but not by μCT imaging.

#### UTE measured bound water to assess bone organic matrix density

**Figure 12:**
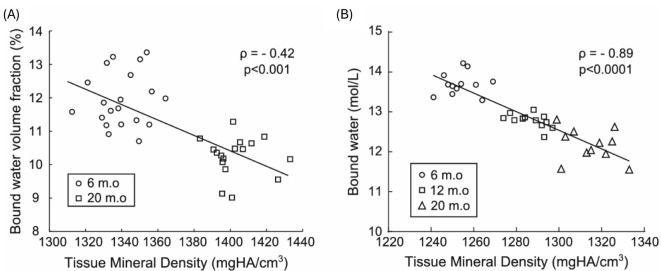
Bound water decreases as mineralization increases in rodents throughout life, as evidenced in mice **(A)** and rats **(B)**, where bound water was calculated as the volume fraction of the bone tissue volume (%) or as the concentration of protons (mol/L) in the bone tissue volume in which μCT determined the latter. Spearman’s rank correlation was performed to calculate the correlation coefficient (ρ). From Ref. (113) with permission.

UTE MRI can map bound water in cortical and trabecular bone (21, 23, 60). Bound water is a surrogate of bone organic matrix density and negatively correlates with bone mineral density, as shown in **Figure 12** (113). It is also reported that bound water in human cortical bone decreases with age, although osteonal remodeling throughout life with only modest changes in tissue mineral density or ash fraction with age after skeletal maturation (114). Bound water and bone density are directly correlated with human cortical bone’s material strength (72, 84, 111, 112).

#### UTE-MT measures to assess bone mechanical properties

**Figure 13:**
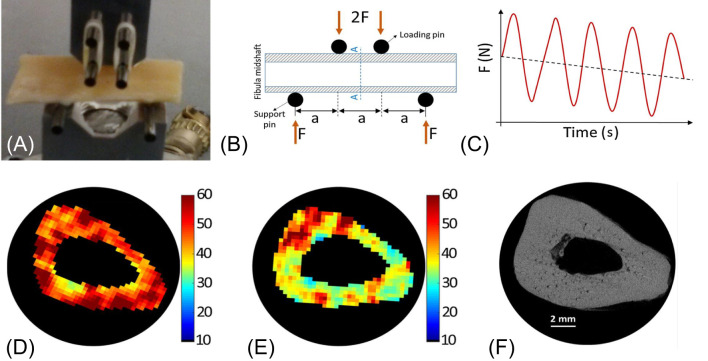
A representative 4-point bending setup and force-time diagram **(A-C)**, as well as MMF maps before **(D)** and after **(E)** loading with marked changes but little change in µCT image and porosity map **(F)**. From Ref. (73) with permission.

UTE-MT can indirectly assess collagen backbone protons, providing information about cortical porosity and mechanical properties (73, 81–83). A recent study reported a moderate to strong negative correlation between UTE magnetization transfer ratio (MTR) and μCT porosity (R^2^ = 0.46–0.51), while a moderate positive correlation was observed between MTR and yield stress (R^2^ = 0.25–0.30) and failure stress (R^2^ = 0.31–0.35).A weak positive correlation (R^2^ = 0.09–0.12) between MTR and Young’s modulus at all off-resonance saturation frequencies was also observed (115). UTE-MT measured MTR provides quantitative information on cortical bone and is sensitive to μCT porosity and biomechanical function. MMF derived from UTE-MT imaging can assess mechanical failures after bone stress injury, which is difficult to evaluate using other techniques (83). In another study (73), fibular samples (n=14) were subject to cyclic loading using a 4-point bending setup (**Figure 13**). Loading was applied to reduce bone stiffness by 20%. Then, bone samples were imaged with UTE MRI and μCT before and after loading. MMF from two-pool UTE-MT modeling decreased by 12% on average, while μCT porosity measured at 6 μm voxel size showed no significant change. A representative sample is shown in **Figure 13**, with averaged MMF decreasing from 63% to 55% (p=0.0001), but no detectable changes in μCT porosity (73).

#### UTE biomarkers for comprehensive assessment of bone and fracture risk

In recent years, many studies have shown that UTE MRI can provide markers of cortical bone porosity, morphologic structure, mineralization, and osteoid density, which are useful measures of bone health (20–23, 76, 84–91, 116–120). In a recent study, Jones et al. reported UTE MRI of 15 participants with OP and 19 without OP (117). The OP group showed elevated pore water (11.6 mol/L vs. 9.5 mol/L; *P* = 0.007) and total water densities (21.2 mol/L vs. 19.7 mol/L; *P* = 0.03), and lower cortical bone thickness (4.8 mm vs. 5.6 mm; *P* < 0.001) and ^31^P density (6.4 mol/L vs. 7.5 mol/L; *P* = 0.01) than the non-OP group, respectively. Meanwhile, there was no evidence of a difference in bone water (BW) or ^31^P-to-BW concentration ratio. Furthermore, pore and total water densities were inversely associated with DXA and HR-pQCT measured BMD (*P* < 0.001) (117). In another study, Jerban et al. investigated the differences in water and collagen contents in tibial cortical bone between female osteopenia (OPe) patients, osteoporosis (OPo) patients, and young participants (Young) using a clinical 3T scanner (91). They found MMF, BWPD, and MMPD were significantly lower in OPo patients than in the young group, whereas T1, TWPD, and PWPD were significantly higher in OPo patients. The largest OPo/Young average percentage differences were found in MMF (41.9%), PWPD (103.5%), and MMPD (64.0%), with PWPD significantly higher (50.7%), while BWPD significantly lower (16.4%) in OPe than the Young group on average. Meanwhile, MMF was significantly lower (27%) in OPo patients compared with OPe group (91). As a result, UTE-MRI measured TWPD, PWPD, and MMF were recommended to evaluate individuals with OPe and OPo. Manhard et al. also demonstrated the feasibility of quantitatively mapping bound and pore water *in vivo* in human cortical bone with practical human MR imaging constraints (84). Jacobson et al. reported a comprehensive set of UTE MRI biomarkers to assess cortical bone. They found the UTE MRI-derived porosity index and signal-intensity-based estimated BMD correlated with the HR-pQCT variables (porosity: *r* = 0.73, *p* = 0.006; BMD: *r* = 0.79, *p* = 0.002) (120).

**Figure 14:**
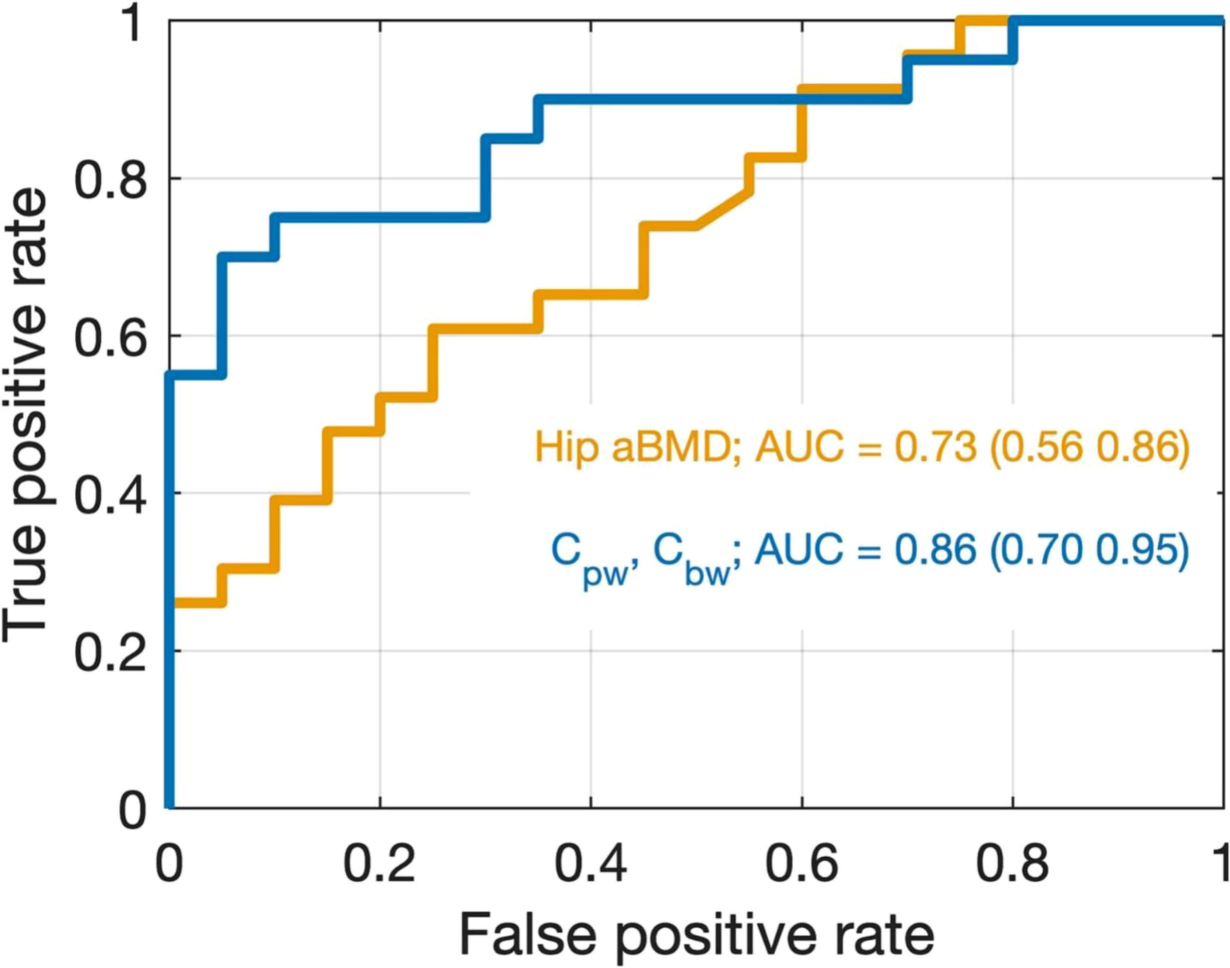
Receiver operating characteristic (ROC) curves for discriminating between non-fracture and distal radius fracture cases using two logistic regression models. The model in orange uses only hip BMD as a predictor which was the best model found without the inclusion of UTE MRI data. The model in blue uses both C_pw_ and C_bw_ as predictors, which was the best overall model. Although the 95% CIs of the AUCs overlap, the data are trending toward the conclusion that the UTE MRI better discriminates Fx from Non-Fx patients than does DXA in the present study. From Ref. (121) with permission.

UTE MRI has also been used to assess fracture risk. In a recent study, Nyman et al. quantified bound water concentration (C_bw_) and pore water concentration (C_pw_) in the radius and tibia as predictors of bone fragility (121). Maps of C_bw_ and C_pw_ were acquired from the uninjured distal third radius of 20 patients who experienced a fragility fracture of the distal radius (Fx) and 20 healthy controls (Non-Fx), and from the tibia mid-diaphysis of 30 women with clinical OP (low T-scores) and 15 women without OP (normal T-scores). They found C_bw_ was significantly lower (p = 0.0018) and C_pw_ was higher (p = 0.0022) in the Fx group than in the Non-Fx group. The area-under-the-receiver operator characteristics curve (AUC with 95% confidence intervals) was 0.73 (0.56, 0.86) for hip BMD (best predictors without MRI) and 0.86 (0.70, 0.95) for the combination of C_bw_ and C_pw_ (best predictors overall), as shown in **Figure 14**. Meanwhile, C_bw_ was significantly lower (*p* = 0.0005) in women with OP (23.8 ± 4.3 ^1^H mol/L) than in women without OP (29.9 ± 6.4 ^1^H mol/L). They also found that it was C_bw_, not C_pw_, which was sensitive to bone-forming osteoporosis medications over 12 months. Their results are largely consistent with the study by Gallant et al. (122), who found the hydroxyl groups on raloxifene provided a possible explanation for the therapeutic effect of raloxifene, a Food and Drug Administration (FDA)-approved agent that is designed to treat bone loss, decrease fracture risk, and improve bone mechanical properties. The benefits of raloxifene treatment are essentially independent of bone mass changes and are mediated by an increase in matrix-bound water as measured by UTE MRI. The study suggests a cell-independent mechanism that can be utilized for novel pharmacological approaches to enhancing bone strength (122).

#### Contrast-enhanced UTE to monitor fracture repair

**Figure 15:**
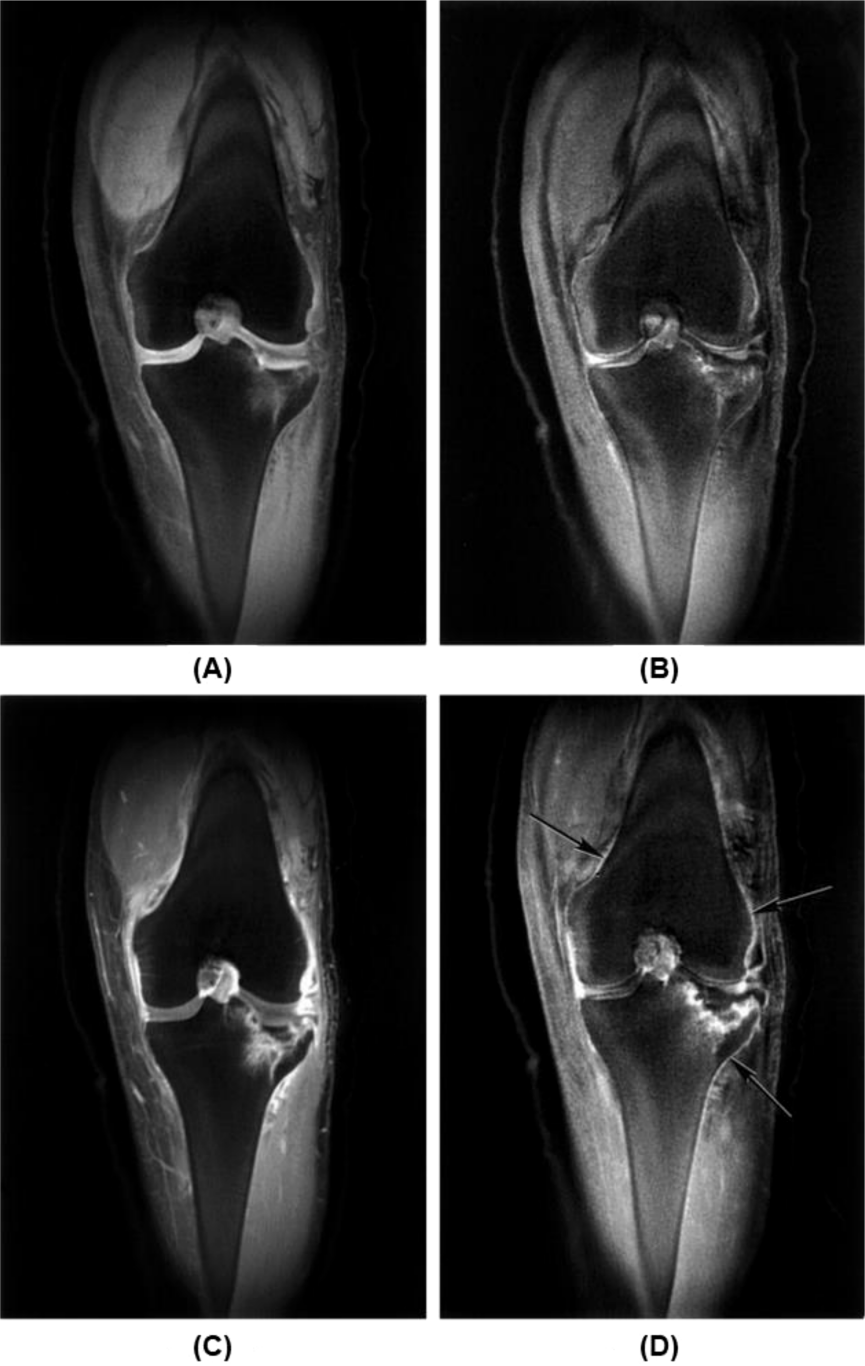
Fracture of tibial plateau 2 days after injury is seen with coronal fat-suppressed UTE (TR/TE=500/0.08 ms) **(A)** and echo subtraction (TE=0.08 minus TE=17.7 ms) **(B)** images before enhancement and the corresponding images **(C, D)** after enhancement, with extensive enhancement in blood vessels in **(C)** and specific enhancement of the periosteum in **(D)**. From Ref. (104) with permission.

Bone is highly vascularized. Perfusion plays an important role in the growth and development of bone as well as in disease and healing (99–102). Reduced perfusion is observed in the trabecular bone of patients with OP (98). It is believed that decreased osseous vascularity contributes to increased fracture risk (123). Reduced perfusion occurs in synchrony with reduced BMD in vertebral trabecular bone (124). UTE can be used to evaluate bone perfusion (104, 105). There is an extensive enhancement in blood vessels due to fracture of the tibial plateau two days after injury, with specific enhancement of the periosteum distinguished from that of blood vessels, as shown in **Figure 15** (104). Even without contrast enhancement, UTE can detect callus formation from a 22-year-old male with a fractured tibia examined 3 weeks after injury (125).

## Discussion

UTE-MRI techniques offer significant advancements in assessing cortical and trabecular bone properties, providing valuable insights beyond traditional imaging methods, such as DXA, CT, HR-pQCT, ultrasound, and conventional MRI (24–26). High signal and contrast can be created for cortical and trabecular bone through a series of contrast mechanisms outlined in this review article. Techniques like ZTE MRI offer a radiation-free alternative for generating CT-like bone contrast. A series of quantitative UTE MRI techniques are also introduced. The ability to quantify total, bound, and pore water content has shown strong correlations with bone microstructure, mechanical properties, and age-related changes, making them promising biomarkers for evaluating fracture risk and osteoporosis. More advanced techniques, such as UTE-QSM and UTE-MT (72, 73, 77–83, 92–98, 126–128), enable us to evaluate bone mineral content and organic matrix density. Dynamic UTE imaging provides information about bone perfusion and modeling and can be used to monitor fracture healing (104, 105).

The UTE MRI techniques may provide new opportunities in assessing bone properties and fracture risk in not only osteoporosis but also other metabolic diseases such as osteopenia, osteomalacia, Paget’s disease, hypophosphatasia, chronic kidney disease–mineral and bone disorder, diabetes, etc. For example, type 2 diabetes (T2D) is characterized by normal or high BMD but impaired bone strength (129–131). Animal and specimen studies indicate that brittle behavior in T2D bone is primarily due to a substantial reduction in collagen capacity for deformation (132–138). High glucose levels lead to the creation of advanced glycation end-products (AGEs), which cause non-enzymatic crosslinking, thereby increasing brittleness of the otherwise elastic collagen fibers and reducing bone toughness (132–138). Quantitative magnetization transfer MRI has been extensively studied to probe extracellular matrix (ECM) and measure the crosslinking of collagen and other polymers (139–141). UTE-MT modeling can measure collagen backbone proton fraction and exchange rates between water and collagen protons (73, 78–83). The exchange rates can be used to assess collagen crosslinking and potentially explain the impaired bone strength in T2D (137, 142).

This review has several limitations. First, the review summarized solid-state 1H UTE techniques. 31P UTE MRI techniques and their applications were only briefly mentioned without systematic discussion. Second, the review only discussed applications in OP. The UTE MRI techniques can also be applied to other metabolic bone diseases.

## Conclusion

With a decade of technical development, the advanced UTE-type MRI sequences allow direct imaging of bone with high signal and contrast. Quantitative UTE MRI techniques can assess all the major components of bone, including water, collagen, and mineral. Advanced UTE techniques can map different bone water components (total water, bound water, and pore water) and evaluate bone perfusion. UTE sequences can also assess bone microstructure, including cortical porosity and trabecular structure. UTE MRI can map phosphorus content, assess bone mineral density, and differentiate between mature and newly remodeled bone. In summary, UTE MRI provides a comprehensive package to assess all bone components (mineral, collagen, water) and microstructure (cortical porosity, trabecular microstructure) using a single modality for improved detection of bone deficits, with potential advantages over conventional X-ray based techniques which can only assess bone mineral. Further research is needed to establish the clinical significance of these UTE-type MRI techniques.
